# Diet-Derived Circulating Antioxidants and Risk of Stroke: A Mendelian Randomization Study

**DOI:** 10.1155/2022/6457318

**Published:** 2022-01-17

**Authors:** Rujia Miao, Jing Li, Changjiang Meng, Yalan Li, Haibo Tang, Jie Wang, Peizhi Deng, Yao Lu

**Affiliations:** ^1^Health Management Center, The Third Xiangya Hospital, Central South University, Changsha, China; ^2^Department of Rehabilitation, The Second Xiangya Hospital, Central South University, Changsha, China; ^3^Department of Cardiology, The Third Xiangya Hospital, Central South University, Changsha, China; ^4^Clinical Research Center, The Third Xiangya Hospital, Central South University, Changsha, China; ^5^Department of Metabolic and Bariatric Surgery, The Third Xiangya Hospital, Central South University, Changsha, China; ^6^School of Life Course Sciences, King's College London, London, UK

## Abstract

**Background:**

Oxidative stress is crucial in stroke pathogenesis. Many cohort-based studies suggested that the intake of exogenous antioxidants originated from food may prevent stroke. However, the corresponding randomized controlled trials did not show diet-derived antioxidants have a protective effect on stroke.

**Objectives:**

To examine the association of genetically proxied diet-derived antioxidants with stroke risk using Mendelian randomization.

**Methods:**

We performed a two-sample Mendelian randomization (MR) analysis to evaluate the causal effect of diet-derived antioxidants on stroke risk. For exposure data, we extracted genetic variants as instrumental variables (IVs) that are strongly associated with frequently used diet-derived antioxidants, including vitamin C, vitamin E (*α*-tocopherol, *γ*-tocopherol), carotene, retinol, zinc, and selenium, from a large-scale genome-wide association study (GWAS). We obtained IVs' corresponding effect estimates on the risk of total stroke and ischemic stroke from a GWAS meta-analysis with 40,585 cases and 406,111 controls. Finally, we applied five types of Mendelian randomization analysis to obtain preliminary MR results and performed four three kinds of sensitivity analysis to verify them.

**Results:**

According to the primary MR estimations and further sensitivity analyses, we established two robust associations after Bonferroni correction: genetically proxied circulating *γ*-tocopherol was causally associated with total stroke [odds ratio (OR) = 0.68, 95% confidence interval (CI) (0.52-0.88), *p* = 3.78*E* − 03] and ischemic stroke [OR = 0.66, 95% CI (0.51-0.86), *p* = 2.34*E* − 03]. There was no evidence to support the causal effect of other diet-derived antioxidants on the risk of total stroke and ischemic stroke.

**Conclusion:**

Our study revealed a protective impact of genetic susceptibility to high circulating *γ*-tocopherol levels on stroke risk, providing new information on the potential therapeutic targets for primary stroke prevention.

## 1. Introduction

According to World Health Organization, stroke has become the second-leading cause of total death and the third leading cause of disability worldwide. In 2019, there were 12.2 million incident cases and 101 million prevalent cases of stroke globally, where ischemic stroke accounted for 62.4%. Evidence-based prevention strategies by reducing the exposure to stroke risk factors were vital for relieving the burden of public health. Currently, the established top five risk factors for stroke are high systolic blood pressure, body mass index, fasting plasma glucose, ambient particulate matter pollution, and smoking [1]. Notably, in addition to these conventional risk factors, oxidative stress associated with excessive production of reactive oxygen species (ROS) is involved in the pathogenesis of stroke [2]. High concentrations of ROS like prooxidant exceeds the counterbalance ability of antioxidants, resulting in cerebrovascular impairment through cellular and vascular mechanism, including endothelial dysfunction, platelet aggregation, and atherosclerosis [3]. It is not surprising that nonselective antioxidants are potentially protective in stroke prevention via scavenging excessive ROS. In fact, it has been reported that endogenous antioxidant substances, such as bilirubin, are causally associated with decreased stroke risk [4]. Many cohort-based studies on stroke prevention support our hypothesis that stroke-susceptible individuals will benefit from regular intake of diet-derived antioxidants [5–7]. Studies suggested zinc and selenium, as antioxidants and anti-inflammatory agents, were inversely associated with the incidence of stroke [8–10]. Conversely, many randomized controlled trials (RCTs) and their meta-analyses have failed to demonstrate that supplementary antioxidants reduce the incidence of stroke [11–13]. It is unclear whether the protective effect of antioxidants on stroke incidence in observational studies was attributed to the bias by confounders such as subjects who preferred healthy diets containing multiple antioxidants or were stuck to a healthier lifestyle. These confounding factors potentially affected cerebrovascular condition. Moreover, reverse causation between diet-derived antioxidants and stroke should also be considered.

To overcome the bias from previous studies, we improve the research design by applying Mendelian randomization (MR) analysis that exploited genetic variants as instrumental variables to establish a strong causal inference between exposure levels of common diet-derived antioxidants and risk of total and ischemic stroke without involving confounders and reverse causations [14].

## 2. Method

### 2.1. Study Design

**Figure 1:**
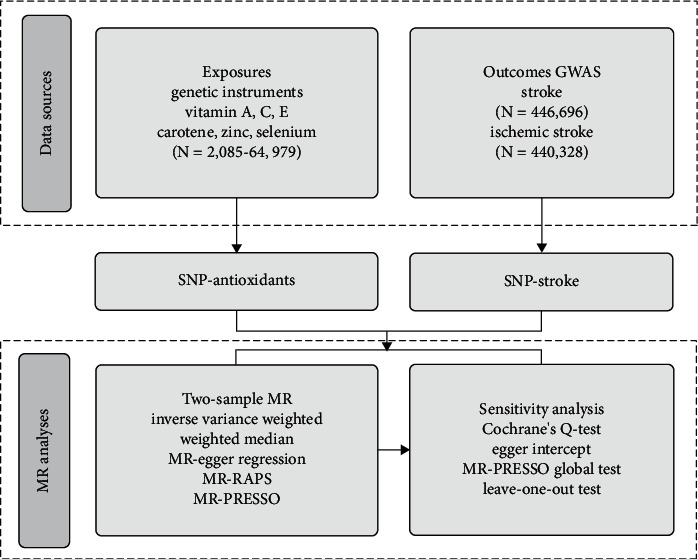
Diagram of Mendelian randomization framework in this study. SNPs for dietary antioxidants (vitamin A, vitamin E, vitamin C, carotene, zinc, and selenium) were identified as genetic instrumental variables. Summary statistics for gene-stroke or gene-ischemic stroke associations were obtained from UK Biobank. For each exposure, MR analyses (primary analysis using inverse-variance weighted (IVW), weighted median, MR-Egger regression, MR-RAPS, MR-PRESSO, and sensitivity analyses using Cochrane's test, Egger intercept, MR-PRESSO global test, and leave-one-out test) were performed. GWAS: genome-wide association study; SNP: single-nucleotide polymorphism; MR: Mendelian randomization.

The general design of the current study is illustrated in Figure 1. First of all, we obtained available genetic variants from the large-scale GWASs for vitamin C (ascorbate), vitamin E (*α*-tocopherol), vitamin E (*γ*-tocopherol), carotene, vitamin A (retinol), zinc, and selenium. Secondly, we selected the summary data of stroke and ischemic stroke from a GWAS meta-analysis. Finally, the causal relationships between diet-derived antioxidants and stroke risk were assessed by a two-sample MR analysis and several sensitivity analyses.

### 2.2. Data for Exposures

Our primary exposures were genetically determined diet-derived antioxidants. In this study, a total of seven diet-derived antioxidants were considered: vitamin C (ascorbate), vitamin E (*α*-tocopherol), vitamin E (*γ*-tocopherol), carotene, vitamin A (retinol), zinc, and selenium. We identified single-nucleotide polymorphisms (SNPs) associated with these diet-derived antioxidants as IVs [*p* < 5 × 10^−6^; linkage disequilibrium (LD); *r*^2^ < 0.001, LD distance > 10,000 kb] from the large-scale GWASs (sample sizes ranging from 2,085 to 64,979) [15, 16]. The strength of the correlation between SNP and diet-derived antioxidants was expressed as an *F*-statistic. Overall, *F* − statistic > 10 suggested a strong correlation between the IVs and antioxidants.

### 2.3. Data for Outcomes

We selected total stroke and ischemic stroke as the main outcomes in our study. Summary statistics on the association of exposure-related SNPs with outcomes were abstracted from a large meta-analysis including 17 studies for European participants. The dataset involved 40,585 total stroke cases, 34,217 ischemic stroke cases, and 406,111 controls [17]. To our knowledge, there was no sample overlap between the exposure and outcome GWASs.

### 2.4. Statistical Analysis

#### 2.4.1. Two-Sample Mendelian Randomization Analysis

We used the classical MR model to examine the causal relationships between these diet-derived antioxidants and the stroke risk (Figure S1). The selection of IVs includes the following criteria: (i) the IVs must be closely associated with diet-derived antioxidants (in this study, defined as the genetic association *p* < 5 × 10^−6^); (ii) not related to confounders of antioxidants and stroke (we conducted a phenome-wide association test to evaluate the relationship between IVs and potential confounders such as body mass index, blood pressure, plasma lipid levels, hypertension, and smoking using PhenoScanner V2 [18]; and (iii) affect stroke only through these antioxidants. In our MR analysis, we used five different methods [inverse-variance weighted (IVW), weighted median, MR-Egger regression, MR-robust adjusted profile score (MR-RAPS), and MR-Pleiotropy Residual Sum and Outlier (MR-PRESSO)] to test the association between dietary antioxidants and stroke or ischemic stroke. The IVW provides MR estimation by combining each Wald ratio of multiple SNPs, showing the largest statistical power among all MR methods [19]. If more than 50% of the weight comes from valid genetic variation, the weighted median could provide accurate estimations [20]. The MR-Egger regression mainly detects and explains horizontal pleiotropy [21]. Furthermore, we also used MR-PRESSO to detect potential outliers and adjust pleiotropy by removing outliers if necessary [22]. Since we used a relatively higher significant threshold (*p* < 5 × 10^−6^) to select genetic variants, we further performed the MR-RAPS to obtain MR estimations using potentially weak instruments [23].

#### 2.4.2. Sensitivity MR Analyses

In this study, Cochrane's *Q* test [24], Egger regression intercepts [25], and MR-PRESSO global test were used for sensitivity analysis to further examine heterogeneity and horizontal pleiotropy. Cochrane's *Q* test was applied to quantify heterogeneity across instrumental variables. MR-Egger intercept tests were used to describe the potential horizontal pleiotropy in the analysis. In addition, we performed a leave-one-out test by sequentially removing each SNP and reestimated the MR results. Based on the above analyses, we took IVW as the primary causal effect estimates and considered the consistency across all MR methods.

MR analysis was performed in R (version 4.0.3) with R packages “TwoSampleMR”, “MR-raps”, and “MR-PRESSO”. The multiple comparisons adjusted *p* value < 0.0007 (0.05/7) after Bonferroni correction was thought to be statistically significant.

## 3. Results

### 3.1. Exposure and Outcome

The characteristics of the participants from antioxidants and stroke datasets are shown in Table 1. We exhibited SNPs associated with vitamin C (ascorbate), vitamin E (*α*-tocopherol), vitamin E (*γ*-tocopherol), carotene, vitamin A (retinol), zinc, and selenium in Table S1. Totally, we enrolled 61 SNPs as IVs for seven antioxidants (*F*-statistics: 20.75-152.28).

### 3.2. Main Findings

**Figure 2:**
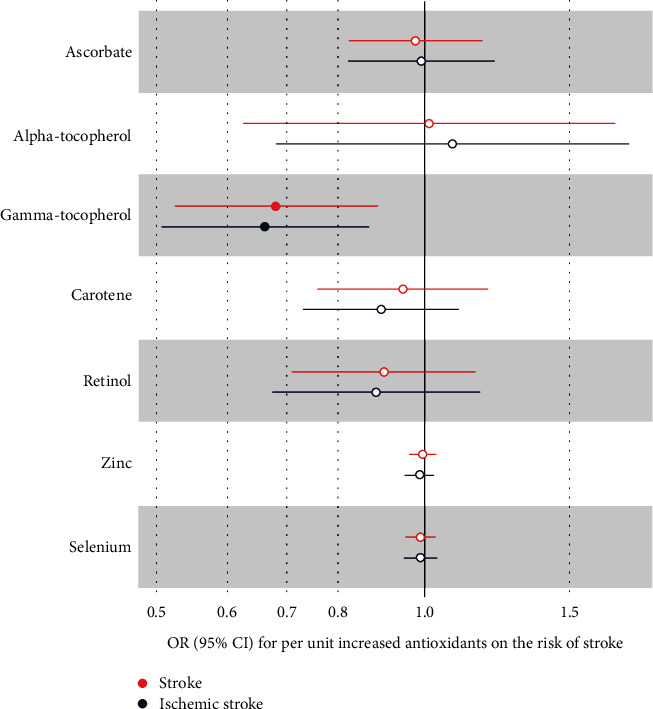
The association between genetically determined diet-derived antioxidants and the risk of stroke. Estimated ORs (odds ratio) for the effect of per unit increase in ascorbate, alpha-tocopherol, gamma-tocopherol, carotene, retinol, zinc, and selenium on stroke and ischemic stroke obtained from an inverse-variance weighted (IVW) analysis.

Table 2 and Figure 2 show MR results for the effects of diet-derived antioxidants on stroke and ischemic stroke. There was clear evidence that genetically determined blood *γ*-tocopherol level was causally associated with total stroke [OR = 0.68, 95% CI (0.52-0.88), *p* = 3.78*E* − 03] and ischemic stroke [OR = 0.66, 95% CI (0.51-0.86), *p* = 2.34*E* − 03]. The results from other MR methods showed good consistency with IVW (*p* < 0.05 in weighted median and MR-RAPS). Although we did not find a potent connection between *γ*-tocopherol and total stroke [OR = 0.80, 95% CI (0.21-3.03), *p* = 7.57*E* − 01] or ischemic stroke [OR = 0.67, 95% CI (0.19-2.45), *p* = 5.75*E* − 01] in MR-Egger regression, we observed that estimates had similar magnitude and direction with other MR methods. There was little evidence of causal effects for other diet-derived antioxidants on total stroke and ischemic stroke risk.

### 3.3. Sensitivity Analysis

Cochrane's *Q* test showed that only in vitamin C (ascorbate) did significant heterogeneity exists with total stroke and ischemic stroke (all *p* < 0.05 in IVW and MR-Egger regression). There was no evidence of directional pleiotropy existing for all antioxidants according to MR-Egger intercept and MR-PRESSO global test except for the association between vitamin C and total stroke (*p* < 1.00*E* − 03) and ischemic stroke (*p* = 1.00*E* − 03) (Table 3). Besides, we identified rs68344631 as an outlier SNP (*p* < 1.00*E* − 02) in the MR-PRESSO outlier test, and then, we found that the outlier-corrected result had a similar range and direction with raw analyses (Table S2). We further performed a phenome-wide association analysis using PhenoScanner V2, suggesting that rs261301 (an IV of *γ*-tocopherol) was associated with lipoprotein and cholesterol (*p* = 4.39*E* − 24). However, we did not find there were any IVs connected with body mass index, blood pressure, smoking, and hypertension (Table S4). Therefore, we recalculated the MR estimation by removing the invalid SNP and found that the association between *γ*-tocopherol and total stroke [OR = 0.68, 95% CI (0.50-0.93), *p* = 1.53*E* − 02] and ischemic stroke [OR = 0.66, 95% CI (0.49-0.89), *p* = 6.40*E* − 03] kept consistent with primary analysis (Table S3). Finally, in the leave-one-out analyses, we found that the risk estimates of genetically predicted antioxidants in diet and risk of stroke or ischemic stroke kept consistent substantially after excluding one SNP at each time (Figure S2 and Figure S3).

## 4. Discussion

The current MR study demonstrated that genetically proxied higher circulating *γ*-tocopherol levels were causally associated with total and ischemic stroke, but we were unable to find significant associations between genetically higher exposures of other typical antioxidants levels (circulating ascorbate, *α*-tocopherol, carotene, retinol, zinc, and selenium) and stroke/ischemic stroke.

An RCT explored the preventive effect of vitamin E on ischemic stroke when it was used alone, and the outcome remained inconclusive [12]. When exploring the effect of isomer products of vitamin E, a former study found that serum *γ*-tocopherol was inversely associated with ischemic stroke in men from a large-scale cohort [26]. In addition to the effect of subtypes, we should notice that genetic predisposition affected the entire life cycle, whereas supplementation only worked during the trial. A low-dose lifelong exposure may accumulate stronger potential biological effects than the temporary high-dose supplements when a long period of time is needed to develop stroke. To our knowledge, this was the first robust causal evidence that *γ*-tocopherol could be potentially applied in stroke prevention. With genetic variants as instrumental variables, MR overcame the limitation of considerable confounding factors in observational studies and provided a more precise estimation of causality. A few RCTs suggested that short-term supplementation of a *γ*-tocopherol-rich mixture of tocopherols restored vascular endothelial function from hyperglycemia and smoking-induced impairment [27, 28], implying the translational value of *γ*-tocopherol in early-stage protection against subsequent atherosclerosis and vascular disease. As to the finding that no effect of *α*-tocopherol was observed, it might result from a paradoxical effect of *α*-tocopherol on oxidation. As the most active form of vitamin E in human body, previous studies have reported that excessive concentrations of *α*-tocopherol can cause oxidative stress, leading to lipid peroxidation mediated by the tocopherol radicals [29].

The robust null results of circulating ascorbate, carotene, retinol, zinc, and selenium in our study suggested that kinds of long-term exposure to higher levels of antioxidant did not reduce the risk of stroke, which was consistent with early findings from the large-scale RCT and meta-analyses on trials [11, 30–32]. However, there were always exceptions: an RCT [33] found that taking the *β*-carotene supplement alone modestly decreased the incidence of cerebral infarction among men with greater alcohol consumption; the case-control trial showed a significant inverse association between plasma retinol and the risk of the first time stroke among Chinese hypertensive adults [34]. These were inconsistent with our results and might be due to the underlying fact that the characteristics of the population included in the MR and RCT did not coincide. Therefore, we assumed that the use of antioxidants was supposed to be selective, at least according to the gender and existing cardiovascular risks, and further population categories should be done. As previously reported similar cases, implementation of MR could be an informative step in the assessment of antioxidant as a chemoprotection targeting cardiovascular diseases: three large databases for genetically predicted antioxidants (*α*-, *γ*-tocopherol, retinol, ascorbate, and carotene) and coronary heart disease (CHD) associations were MR-analyzed; the results did not support the protective effect of these diet-derived antioxidants on CHD risk [35], suggesting the limited CHD prevention benefit from antioxidant supplement. Another MR study indicated that higher circulating vitamin E level might increase the risk of CHD and myocardial infarction [36], thus promoting the reassessment of the safety and efficacy of vitamin E supplementation. Genetically instrumented zinc [37] but not selenium [38] was positively associated with CHD; this implied the underlying risk of microelements with antioxidative capacity in vascular health. For completeness, our outcome revealed the limited value of antioxidants in cerebrovascular protection, except for *γ*-tocopherol as a promising supplement.

*γ*-Tocopherol is the most common form of vitamin E found in plant seeds and the derivatives [39]. Epidemiological evidence [40] had indicated that intake of *γ*-tocopherol but not *α*-tocopherol was significantly inversely associated with the risk of death from cardiovascular disease. Mechanistically, *γ*-tocopherol had certain biologically protective properties: *γ*-tocopherol was potent in enhancing the activity of superoxide dismutase (SOD) and endothelial cell nitric oxide synthase in arterial tissue, and increasing the expression of both manganese/copper SOD in the antioxidative manner [41, 42]; besides, *γ*-tocopherol possessed the capacity of anti-inflammation and antiplatelet aggregation independent of antioxidant activity [41, 43]. Previous studies have reported that *γ*-tocopherol may inhibit lipid peroxidation damage and trap reactive nitrogen species [44]. Cooney et al. demonstrated that NO_2_, also a lipid-soluble species, was sequestered through nitration of *γ*-tocopherol which was superior to *α*-tocopherol in the detoxification of NO_2_ [45]. *γ*-Tocopherol may have the potential to scavenge peroxynitrite in inflamed vascular endothelium, thereby limiting the oxidation of BH4 and helping to preserve effective eNOS activity [46]. With all these potencies, *γ*-tocopherol exerted a comprehensive effect against vascular impairment and atherosclerosis originating from diabetes, smoking, aging, and so on.

The strength of the current study was that our MR analysis reflected the impact of lifelong exposure to higher levels of diet-derived antioxidants, considering long-term risks that may not be modified by short-term supplementation treatment. More importantly, MR did not require subjects to be directly exposed to antioxidants, which means it could be implemented at any point without time and resource requirements as RCTs did, thus reducing the possibility of exposing subjects to unnecessary risks and harms [47].

The limitations of our research are as follows: first, the beneficial effects of antioxidants may still exist in unselected subgroups, especially in those with elevated oxidative levels. Besides, traditional treatment plus antioxidants may have a synergistic benefit on stroke. Second, due to the lack of relevant data, we were not able to perform the next step of stroke subtype analysis. Further stroke subtype analysis is needed to determine the correlation between antioxidants and stroke of various subtypes. Third, though no causal association was detected, the potential for the effect size to be too small for identification cannot be fully excluded. Finally, the genetic variants relied on European samples too much, and this was owing to the lack of corresponding data from Asian populations.

## 5. Conclusion

In summary, we have posted evidence of a causal relationship between circulating diet-derived antioxidant/metabolites levels and decreased risk of total and ischemic stroke. The study provided information that though most null results limit the application of antioxidants in preventing stroke, *γ*-tocopherol is a promising chemoprotection targeting stroke incidence.

## Figures and Tables

**Table 1:** Characteristics of diet-derived antioxidants and stroke datasets.

Exposures	Data source	SNP	*F*-statistic	Sample size	Population
Vit. C (ascorbate)	Shin et al.	10	23.67	2,085	European
Vit. E (*α*-tocopherol)	Shin et al.	5	71.42	7,725	European
Vit. E (*γ*-tocopherol)	Shin et al.	7	55.22	6,226	European
Carotene	MRC-IEU	13	76.98	64,979	European
Vit. A (retinol)	MRC-IEU	8	136.20	62,991	European
Zinc	Evans et al.	8	40.13	2,603	European
Selenium	Evans et al.	6	49.70	2,603	European
Outcomes	Data source	Studies	Cases/controls	Sample size	Population
Stroke	Meta-analysis	17	40,585/406,111	446,696	European
Ischemic stroke	Meta-analysis	17	34,217/406,111	440,328	European

Vit: vitamin; SNP: single-nucleotide polymorphism.

**Table 2:** Two-sample Mendelian randomization estimations showing the effects of diet-derived antioxidants on the risk of stroke.

Outcomes	Exposures	Inverse-variance weighted		Weighted median		MR-Egger		MR-RAPS	
		OR (95% CI)	*p* value	OR (95% CI)	*p* value	OR (95% CI)	*p* value	OR (95% CI)	*p* value
Stroke	Vit. C (ascorbate)	0.98 (0.82-1.16)	7.86*E* − 01	0.88 (0.78-1.00)	5.67*E* − 02	0.87 (0.48-1.59)	6.66*E* − 01	0.93 (0.81-1.06)	2.73*E* − 01
	Vit. E (*α*-tocopherol)	1.01 (0.62-1.64)	9.65*E* − 01	0.80 (0.46-1.38)	4.29*E* − 01	0.62 (0.10-3.70)	6.33*E* − 01	0.99 (0.59-1.66)	9.59*E* − 01
	Vit. E (*γ*-tocopherol)	0.68 (0.52-0.88)	3.78**E** − 03	0.68 (0.48-0.96)	3.09**E** − 02	0.80 (0.21-3.03)	7.57*E* − 01	0.66 (0.50-0.87)	3.38**E** − 03
	Carotene	0.94 (0.76-1.18)	6.08*E* − 01	1.02 (0.78-1.32)	8.91*E* − 01	1.02 (0.63-1.67)	9.24*E* − 01	0.94 (0.76-1.17)	5.80*E* − 01
	Vit. A (retinol)	0.90 (0.71-1.14)	3.80*E* − 01	0.86 (0.63-1.16)	3.14*E* − 01	1.22 (0.63-2.37)	5.74*E* − 01	0.87 (0.69-1.11)	2.64*E* − 01
	Zinc	0.99 (0.96-1.03)	7.55*E* − 01	0.99 (0.95-1.04)	8.15*E* − 01	0.90 (0.80-1.01)	1.18*E* − 01	1.00 (0.96-1.03)	8.32*E* − 01
	Selenium	0.99 (0.95-1.03)	5.60*E* − 01	0.99 (0.95-1.04)	7.91*E* − 01	1.02 (0.93-1.11)	7.55*E* − 01	0.99 (0.95-1.03)	5.71*E* − 01
Ischemic stroke	Vit. C (ascorbate)	0.99 (0.82-1.20)	9.26*E* − 01	0.91 (0.79-1.05)	1.84*E* − 01	0.88 (0.45-1.71)	7.18*E* − 01	0.95 (0.81-1.11)	5.16*E* − 01
	Vit. E (*α*-tocopherol)	1.07 (0.68-1.69)	7.59*E* − 01	0.88 (0.50-1.54)	6.47*E* − 01	0.85 (0.15-4.77)	8.67*E* − 01	1.07 (0.64-1.78)	7.93*E* − 01
	Vit. E (*γ*-tocopherol)	0.66 (0.51-0.86)	2.34**E** − 03	0.67 (0.46-0.98)	3.78**E** − 02	0.67 (0.19-2.45)	5.75*E* − 01	0.65 (0.48-0.87)	3.56**E** − 03
	Carotene	0.89 (0.73-1.09)	2.66*E* − 01	0.92 (0.70-1.20)	5.27*E* − 01	0.97 (0.61-1.54)	8.88*E* − 01	0.88 (0.72-1.07)	2.03*E* − 01
	Vit. A (retinol)	0.88 (0.67-1.15)	3.57*E* − 01	0.86 (0.61-1.20)	3.71*E* − 01	1.14 (0.52-2.49)	7.60*E* − 01	0.85 (0.66-1.09)	2.03*E* − 01
	Zinc	0.99 (0.95-1.02)	4.69*E* − 01	0.99 (0.94-1.04)	5.97*E* − 01	0.88 (0.78-1.00)	9.84*E* − 02	0.99 (0.95-1.03)	5.94*E* − 01
	Selenium	0.99 (0.95-1.03)	6.05*E* − 01	0.99 (0.94-1.04)	6.83*E* − 01	1.00 (0.91-1.11)	9.33*E* − 01	0.99 (0.95-1.03)	6.15*E* − 01

*p* values in bold indicate they achieved the nominal significance (*p* < 0.05). Vit.: vitamin; MR: Mendelian randomization; CI: confidence interval; OR: odds ratio.

**Table 3:** The estimations of heterogeneity and horizontal pleiotropy for MR results.

Outcomes	Exposures	IVW		MR-Egger				MR-PRESSO
		*Q*-statistic	*p* value	*Q*-statistic	*p* value	Egger intercept	*p* value	*p* for global test
Stroke	Vit. C (ascorbate)	30.83	3.16*E* − 04	30.26	1.90*E* − 04	0.008 (-0.033-0.049)	7.07*E* − 01	<1.00*E* − 03
	Vit. E (*α*-tocopherol)	5.90	2.07*E* − 01	2.05	8.43*E* − 01	0.014 (-0.034-0.062)	6.11*E* − 01	2.69*E* − 01
	Vit. E (*γ*-tocopherol)	6.56	3.64*E* − 01	6.48	2.62*E* − 01	-0.005 (-0.042-0.033)	8.16*E* − 01	3.75*E* − 01
	Carotene	19.24	8.30*E* − 02	19.00	6.12*E* − 02	-0.004 (-0.023-0.016)	7.20*E* − 01	9.20*E* − 02
	Vit. A (retinol)	8.07	3.27*E* − 01	6.97	3.24*E* − 01	-0.012 (-0.037-0.012)	3.68*E* − 01	3.60*E* − 01
	Zinc	5.44	6.07*E* − 01	2.15	9.05*E* − 01	0.022 (-0.002-0.045)	1.20*E* − 01	5.43*E* − 01
	Selenium	2.79	7.31*E* − 01	2.38	6.66*E* − 01	-0.006 (-0.024-0.012)	5.56*E* − 01	7.17*E* − 01
Ischemic stroke	Vit. C (ascorbate)	33.50	1.09*E* − 04	32.95	6.29*E* − 05	0.008 (-0.037-0.053)	7.25*E* − 01	1.00*E* − 03
	Vit. E (*α*-tocopherol)	4.74	3.15*E* − 01	4.62	2.02*E* − 01	0.007 (-0.040-0.053)	8.00*E* − 01	3.27*E* − 01
	Vit. E (*γ*-tocopherol)	5.31	5.05*E* − 01	5.31	3.79*E* − 01	-0.001 (-0.037-0.036)	9.78*E* − 01	5.47*E* − 01
	Carotene	12.80	3.06*E* − 01	12.63	2.45*E* − 01	-0.003 (-0.021-0.015)	7.16*E* − 01	3.36*E* − 01
	Vit. A (retinol)	9.05	2.49*E* − 01	8.40	2.10*E* − 01	-0.010 (-0.039-0.019)	5.23*E* − 01	2.88*E* − 01
	Zinc	5.63	5.84*E* − 01	2.31	8.89*E* − 01	0.024 (-0.002-0.049)	1.18*E* − 01	5.10*E* − 01
	Selenium	2.05	8.43*E* − 01	1.93	7.48*E* − 01	-0.003 (-0.022-0.016)	7.52*E* − 01	8.32*E* − 01

Vit: vitamin; MR: Mendelian randomization; IVW: inverse-variance weighted.

## Data Availability

The datasets were derived from sources in the public domain: GWAS (https://gwas.mrcieu.ac.uk) and MR-Base (https://www.mrbase.org/).

## Supplementary Materials

Supplementary Materials Figure S1: instrumental variable (IV) assumptions of Mendelian randomization. Figure S2: scatter plot (A, C, E, G, I, K, M) and leave-one-out test (B, D, F, H, J, L, N) for genetically determined antioxidants and risk of stroke. Figure S3: scatter plot (A, C, E, G, I, K, M) and leave-one-out test (B, D, F, H, J, L, N) for genetically determined antioxidants and risk of ischemic stroke. Table S1: single-nucleotide polymorphisms (SNP) associated with diet-derived antioxidants. Table S2: MR-PRESSO outlier-corrected MR analysis for vitamin C (ascorbate) and risk of stroke and ischemic stroke. Table S3: two-sample Mendelian randomization estimations showing the effects of vit. E (*γ*-tocopherol) on the risk of stroke and ischemic stroke by removing outlier rs261301. Table S4: instrumental variable trait of dietary antioxidants in PhenoScanner V2.
