# Supplementary material for: Diet-Derived Circulating Antioxidants and Risk of Stroke: A Mendelian Randomization Study
Source: Oxid Med Cell Longev. 2022 Jan 17;2022:6457318. doi: 10.1155/2022/6457318 (PMC8786473; doi:10.1155/2022/6457318)
Supplement: Supplementary Materials — Figure S1: instrumental variable (IV) assumptions of Mendelian randomization. Figure S2: scatter plot (A, C, E, G, I, K, M) and leave-one-out test (B, D, F, H, J, L, N) for genetically determined antioxidants and risk of stroke. Figure S3: scatter plot (A, C, E, G, I, K, M) and leave-one-out test (B, D, F, H, J, L, N) for genetically determined antioxidants and risk of ischemic stroke. Table S1: single-nucleotide polymorphisms (SNP) associated with diet-derived antioxidants. Table S2: MR-PRESSO outlier-corrected MR analysis for vitamin C (ascorbate) and risk of stroke and ischemic stroke. Table S3: two-sample Mendelian randomization estimations showing the effects of vit. E (γ-tocopherol) on the risk of stroke and ischemic stroke by removing outlier rs261301. Table S4: instrumental variable trait of dietary antioxidants in PhenoScanner V2. [file 6457318.f1.zip › FigureS1.pptx]

## Slide 1
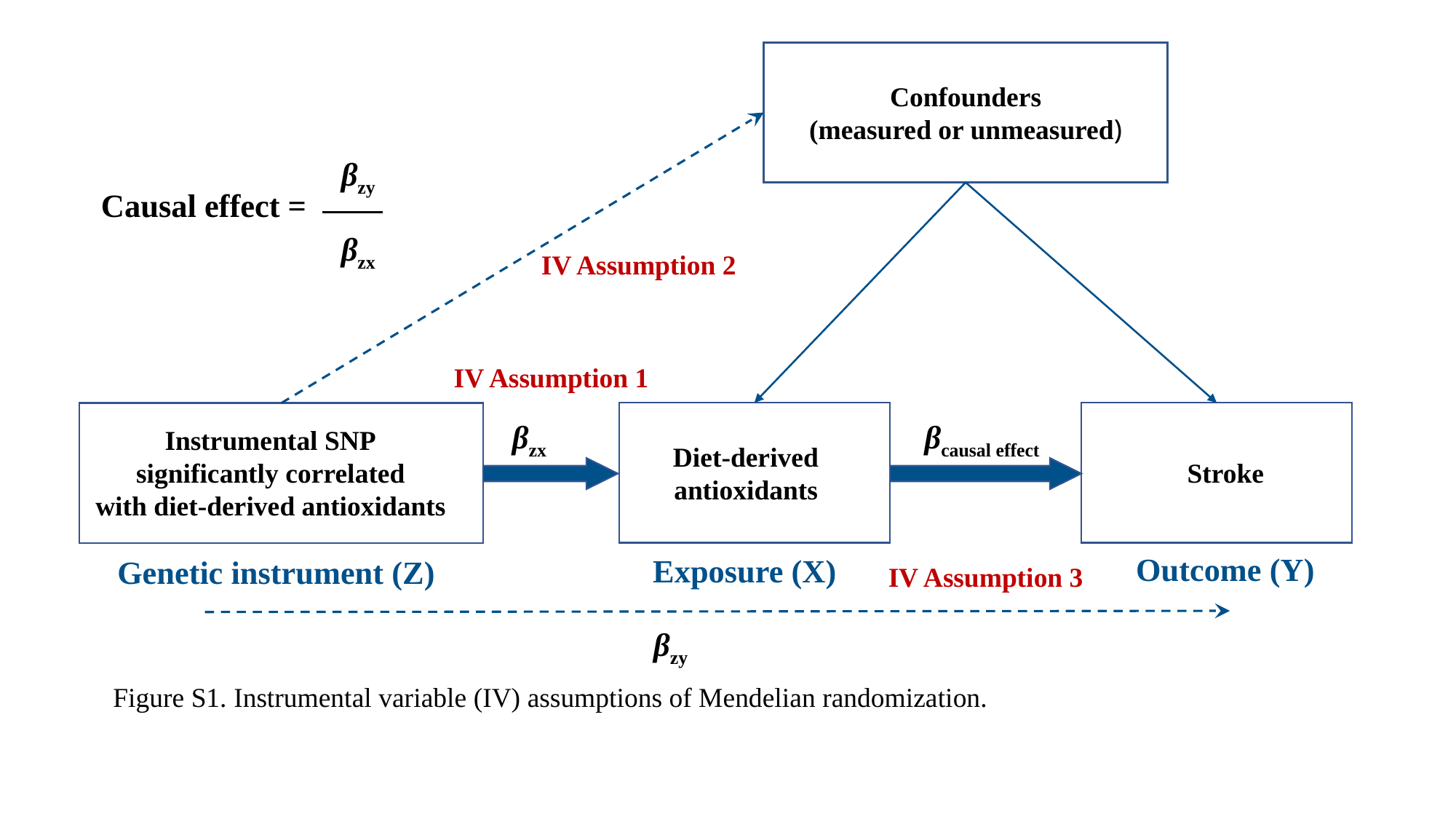

Confounders
(measured or unmeasured)
βzy
Causal effect =
βzx
IV Assumption 2
IV Assumption 1
βzx
βcausal effect
Instrumental SNP
significantly correlated
with diet-derived antioxidants
Diet-derived
antioxidants
Stroke
Outcome (Y)
Exposure (X)
Genetic instrument (Z)
IV Assumption 3
βzy
Figure S1. Instrumental variable (IV) assumptions of Mendelian randomization.
