# Supplementary material for: Diet-Derived Circulating Antioxidants and Risk of Stroke: A Mendelian Randomization Study
Source: Oxid Med Cell Longev. 2022 Jan 17;2022:6457318. doi: 10.1155/2022/6457318 (PMC8786473; doi:10.1155/2022/6457318)
Supplement: Supplementary Materials — Figure S1: instrumental variable (IV) assumptions of Mendelian randomization. Figure S2: scatter plot (A, C, E, G, I, K, M) and leave-one-out test (B, D, F, H, J, L, N) for genetically determined antioxidants and risk of stroke. Figure S3: scatter plot (A, C, E, G, I, K, M) and leave-one-out test (B, D, F, H, J, L, N) for genetically determined antioxidants and risk of ischemic stroke. Table S1: single-nucleotide polymorphisms (SNP) associated with diet-derived antioxidants. Table S2: MR-PRESSO outlier-corrected MR analysis for vitamin C (ascorbate) and risk of stroke and ischemic stroke. Table S3: two-sample Mendelian randomization estimations showing the effects of vit. E (γ-tocopherol) on the risk of stroke and ischemic stroke by removing outlier rs261301. Table S4: instrumental variable trait of dietary antioxidants in PhenoScanner V2. [file 6457318.f1.zip › supplementary-12.12final.docx]

**Supplementary Files**

Figure S1. Instrumental variable (IV) assumptions of Mendelian randomization.

Figure S2. Scatter plot (A, C, E, G, I, K, M) and leave-one-out test (B, D, F, H, J, L, N) for genetically determined antioxidants and risk of stroke

Figure S3. Scatter plot (A, C, E, G, I, K, M) and leave-one-out test (B, D, F, H, J, L, N) for genetically determined antioxidants and risk of ischemic stroke

Table S1. Single nucleotide polymorphisms (SNP) associated with dietary-derived antioxidants

Table S2. MR-PRESSO outlier-corrected MR analysis for vitamin C (ascorbate) and risk of stroke, ischemic stroke

Table S3. Two-sample Mendelian randomization estimations showing the effects of Vit E (γ-tocopherol) on the risk of stroke and ischemic stroke by removing outlier rs261301

Table S4. Instrumental variables trait of dietary antioxidants in PhenoScanner V2

**
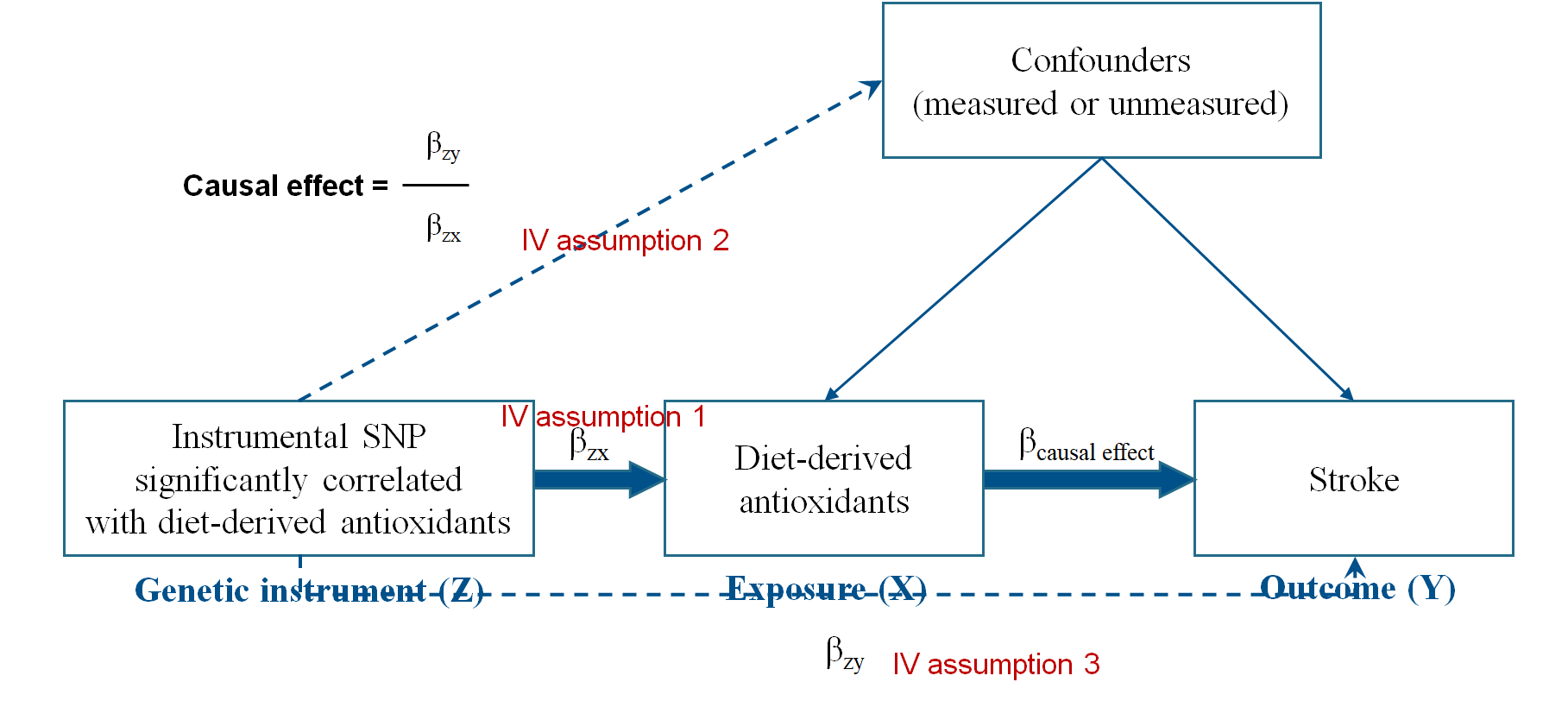
**

**Figure S1. Instrumental variable (IV) assumptions of Mendelian randomization.**

SNP, single nucleotide polymorphism;


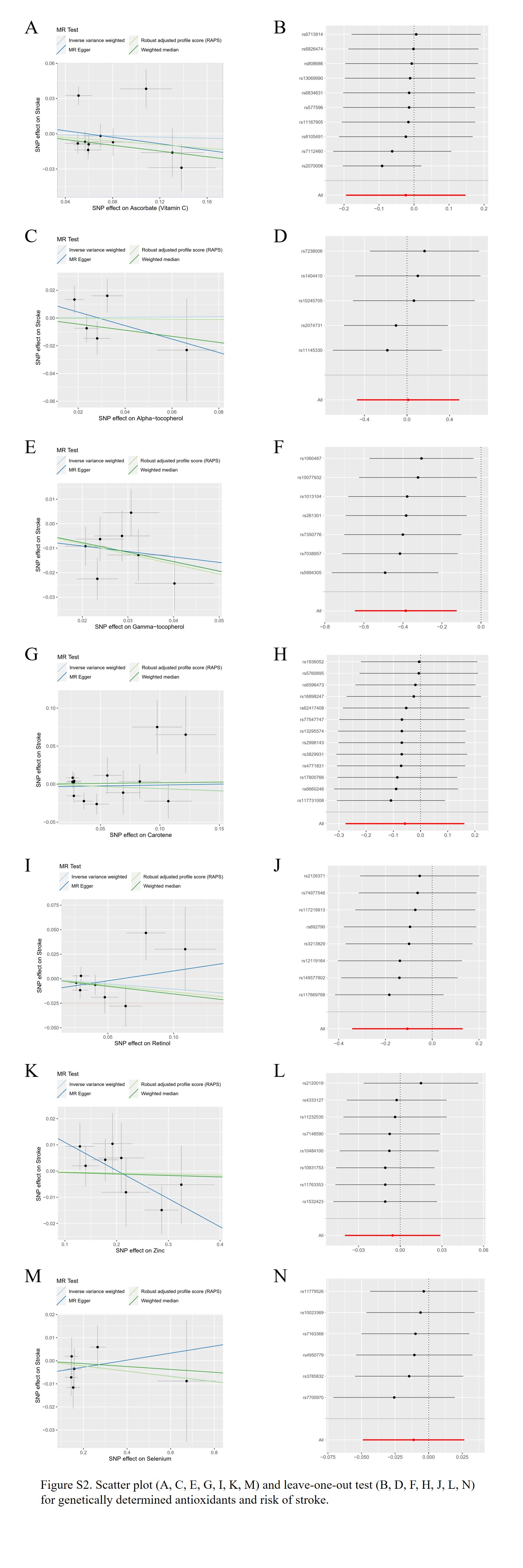


**Figure S2. Scatter plot (A, C, E, G, I, K, M) and leave-one-out test (B, D, F, H, J, L, N) for genetically determined antioxidants and risk of stroke.**


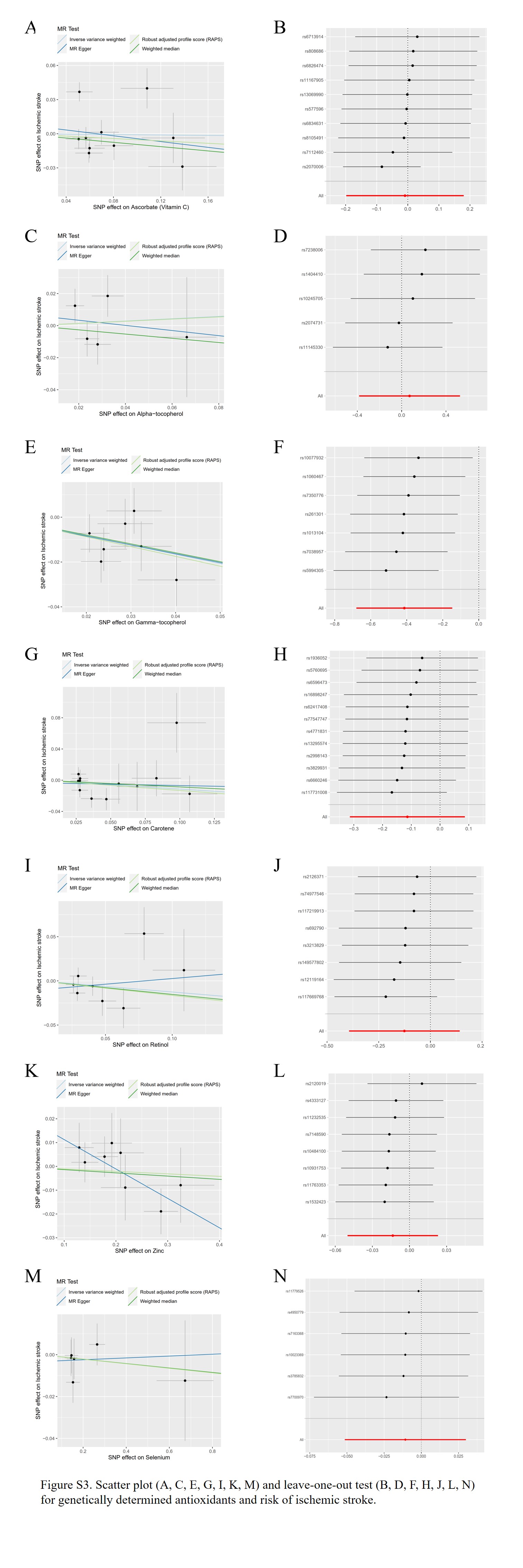


**Figure S3. Scatter plot (A, C, E, G, I, K, M) and leave-one-out test (B, D, F, H, J, L, N) for genetically determined antioxidants and risk of ischemic stroke**

**Table S1. Single nucleotide polymorphisms (SNP) associated with dietary-derived antioxidants**

| SNP | effect allele | other allele | eaf | beta | se | *p*-value | sample size | r2 | F-stat | id.exposure |
| --- | --- | --- | --- | --- | --- | --- | --- | --- | --- | --- |
| rs4950779 | C | T | 0.034 | 0.673 | 0.132 | 3.16E-07 | 2603 | 0.100026 | 48.0877 | ieu-a-1077 |
| rs10023369 | A | G | 0.497 | -0.144 | 0.029 | 4.42E-07 | 2603 | 0.098785 | 47.42603 | ieu-a-1077 |
| rs7700970 | T | C | 0.317 | 0.265 | 0.037 | 7.17E-13 | 2603 | 0.140028 | 70.45075 | ieu-a-1077 |
| rs11779526 | T | A | 0.295 | 0.153 | 0.032 | 1.68E-06 | 2603 | 0.093695 | 44.72977 | ieu-a-1077 |
| rs7163368 | C | T | 0.237 | 0.158 | 0.034 | 4.00E-06 | 2603 | 0.090243 | 42.91838 | ieu-a-1077 |
| rs3785832 | C | T | 0.404 | -0.146 | 0.031 | 1.82E-06 | 2603 | 0.093382 | 44.56473 | ieu-a-1077 |
| rs10931753 | C | G | 0.664 | -0.129 | 0.028 | 4.94E-06 | 2603 | 0.089384 | 31.82783 | ieu-a-1079 |
| rs4333127 | A | G | 0.903 | 0.218 | 0.047 | 3.00E-06 | 2603 | 0.091402 | 32.61836 | ieu-a-1079 |
| rs11763353 | G | A | 0.166 | -0.192 | 0.039 | 6.90E-07 | 2603 | 0.097116 | 34.87678 | ieu-a-1079 |
| rs1532423 | G | A | 0.629 | -0.178 | 0.026 | 6.40E-12 | 2603 | 0.134118 | 50.22374 | ieu-a-1079 |
| rs11232535 | C | T | 0.055 | 0.325 | 0.065 | 6.73E-07 | 2603 | 0.09721 | 34.91429 | ieu-a-1079 |
| rs7148590 | A | G | 0.484 | -0.14 | 0.026 | 1.37E-07 | 2603 | 0.103053 | 37.25399 | ieu-a-1079 |
| rs10484100 | G | A | 0.0895 | -0.209 | 0.045 | 3.30E-06 | 2603 | 0.091019 | 32.46831 | ieu-a-1079 |
| rs2120019 | C | T | 0.208 | -0.287 | 0.033 | 1.55E-18 | 2603 | 0.17102 | 66.89319 | ieu-a-1079 |
| rs1404410 | G | C | 0.2147 | 0.0236 | 0.0052 | 4.57E-06 | 1759 | 0.109042 | 42.90909 | met-a-340 |
| rs10245705 | T | C | 0.0181 | -0.0663 | 0.0127 | 1.95E-07 | 7276 | 0.060967 | 94.40182 | met-a-340 |
| rs11145330 | C | A | 0.1092 | -0.0324 | 0.0068 | 1.95E-06 | 1759 | 0.113187 | 44.74839 | met-a-340 |
| rs7238006 | C | T | 0.0738 | -0.0281 | 0.0057 | 6.77E-07 | 7276 | 0.0582 | 89.85222 | met-a-340 |
| rs2074731 | A | C | 0.1654 | -0.0184 | 0.0039 | 2.31E-06 | 7276 | 0.055354 | 85.20102 | met-a-340 |
| rs6713914 | C | T | 0.4259 | -0.0593 | 0.0116 | 3.22E-07 | 1567 | 0.128656 | 22.9747 | met-a-348 |
| rs13069990 | T | C | 0.3781 | -0.0506 | 0.011 | 4.44E-06 | 2063 | 0.10085 | 23.01544 | met-a-348 |
| rs6834631 | G | T | 0.0404 | -0.1306 | 0.0267 | 1.03E-06 | 2063 | 0.107315 | 24.66829 | met-a-348 |
| rs6826474 | T | C | 0.0393 | -0.1383 | 0.0288 | 1.56E-06 | 2063 | 0.10552 | 24.20704 | met-a-348 |
| rs2070006 | C | T | 0.6287 | -0.0512 | 0.0112 | 4.76E-06 | 2063 | 0.100532 | 22.93478 | met-a-348 |
| rs11167905 | C | T | 0.1455 | -0.0804 | 0.0164 | 9.83E-07 | 2063 | 0.107526 | 24.72259 | met-a-348 |
| rs7112460 | T | C | 0.0658 | 0.1084 | 0.0223 | 1.14E-06 | 2063 | 0.106905 | 24.56273 | met-a-348 |
| rs8105491 | T | G | 0.154 | -0.0698 | 0.0148 | 2.30E-06 | 2063 | 0.103805 | 23.76793 | met-a-348 |
| rs808686 | A | G | 0.6086 | 0.0598 | 0.0128 | 3.01E-06 | 1567 | 0.117655 | 20.74823 | met-a-348 |
| rs577596 | A | G | 0.3267 | -0.0567 | 0.0114 | 6.68E-07 | 2063 | 0.109172 | 25.14743 | met-a-348 |
| rs10077932 | T | C | 0.1376 | -0.0402 | 0.0087 | 4.08E-06 | 5822 | 0.060338 | 53.3329 | met-a-571 |
| rs7038957 | C | T | 0.1688 | 0.0287 | 0.0062 | 3.86E-06 | 5822 | 0.060488 | 53.47447 | met-a-571 |
| rs7350776 | G | C | 0.303 | -0.0239 | 0.0052 | 3.86E-06 | 5822 | 0.060488 | 53.47376 | met-a-571 |
| rs261301 | C | T | 0.8686 | -0.0323 | 0.0068 | 2.06E-06 | 5822 | 0.062167 | 55.05701 | met-a-571 |
| rs1013104 | T | C | 0.4354 | -0.0207 | 0.0045 | 3.83E-06 | 5822 | 0.060509 | 53.49369 | met-a-571 |
| rs1060467 | G | A | 0.4097 | -0.0233 | 0.0045 | 2.61E-07 | 5822 | 0.067432 | 60.05639 | met-a-571 |
| rs5994305 | G | A | 0.1682 | -0.0307 | 0.0062 | 7.15E-07 | 5822 | 0.064913 | 57.65731 | met-a-571 |
| rs1936052 | T | C | 0.155936 | -0.03612 | 0.007668 | 2.50E-06 | 64979 | 0.018469 | 76.39561 | ukb-b-16202 |
| rs6660246 | C | A | 0.450266 | -0.02669 | 0.005531 | 1.40E-06 | 64979 | 0.018927 | 78.32828 | ukb-b-16202 |
| rs12126792 | G | A | 0.011509 | -0.13471 | 0.028005 | 1.50E-06 | 64979 | 0.018873 | 78.10052 | ukb-b-16202 |
| rs77547747 | C | T | 0.056418 | -0.0558 | 0.011834 | 2.40E-06 | 64979 | 0.018501 | 76.53309 | ukb-b-16202 |
| rs6596473 | C | G | 0.299056 | 0.027642 | 0.005976 | 3.70E-06 | 64979 | 0.018153 | 75.06431 | ukb-b-16202 |
| rs62417408 | G | A | 0.037772 | -0.06912 | 0.014655 | 2.40E-06 | 64979 | 0.018501 | 76.53309 | ukb-b-16202 |
| rs16898247 | A | G | 0.018968 | -0.10709 | 0.020031 | 9.00E-08 | 64979 | 0.02097 | 86.96272 | ukb-b-16202 |
| rs13295574 | A | G | 0.303383 | -0.02767 | 0.005982 | 3.70E-06 | 64979 | 0.018153 | 75.06431 | ukb-b-16202 |
| rs3829931 | A | T | 0.973235 | 0.083083 | 0.01779 | 3.00E-06 | 64979 | 0.018322 | 75.77903 | ukb-b-16202 |
| rs17800766 | C | T | 0.011673 | -0.121685 | 0.0254964 | 1.80E-06 | 64979 | 0.018729554 | 77.49579156 | ukb-b-16202 |
| rs2998143 | G | A | 0.603581 | -0.0278722 | 0.00584016 | 1.80E-06 | 64979 | 0.018729554 | 77.49579156 | ukb-b-16202 |
| rs4771831 | A | G | 0.351886 | -0.0265212 | 0.00576207 | 4.20E-06 | 64979 | 0.018049306 | 74.62945018 | ukb-b-16202 |
| rs116995905 | T | C | 0.009812 | -0.132301 | 0.0288061 | 4.40E-06 | 64979 | 0.018011279 | 74.46933182 | ukb-b-16202 |
| rs366337 | G | A | 0.936764 | 0.0543804 | 0.0112221 | 1.30E-06 | 64979 | 0.018984804 | 78.57235875 | ukb-b-16202 |
| rs117731008 | A | G | 0.016818 | 0.0977133 | 0.0212277 | 4.20E-06 | 64979 | 0.018049306 | 74.62945018 | ukb-b-16202 |
| rs5760695 | C | T | 0.086598 | 0.0468481 | 0.0100556 | 3.20E-06 | 64979 | 0.018270179 | 75.55970015 | ukb-b-16202 |
| rs12119164 | G | A | 0.742193 | 0.0295741 | 0.00637063 | 3.40E-06 | 62991 | 0.01850641 | 131.9476781 | ukb-b-17406 |
| rs692790 | C | T | 0.874331 | 0.0404149 | 0.00838208 | 1.40E-06 | 62991 | 0.019223251 | 137.1588161 | ukb-b-17406 |
| rs74977546 | A | G | 0.052896 | -0.063514 | 0.0126784 | 5.50E-07 | 62991 | 0.019952168 | 142.4655549 | ukb-b-17406 |
| rs149577802 | T | C | 0.015116 | -0.108795 | 0.0234115 | 3.40E-06 | 62991 | 0.01850641 | 131.9476781 | ukb-b-17406 |
| rs3213829 | G | T | 0.546482 | 0.025976 | 0.00560984 | 3.60E-06 | 62991 | 0.018459357 | 131.6058859 | ukb-b-17406 |
| rs117669768 | A | G | 0.038236 | 0.0789545 | 0.0147682 | 9.00E-08 | 62991 | 0.021297858 | 152.2833544 | ukb-b-17406 |
| rs2126371 | T | C | 0.317531 | -0.0289453 | 0.00597696 | 1.30E-06 | 62991 | 0.019282015 | 137.5863382 | ukb-b-17406 |
| rs117219913 | C | T | 0.07834 | 0.0476864 | 0.0103813 | 4.40E-06 | 62991 | 0.018293254 | 130.3995917 | ukb-b-17406 |
| rs909570 | A | G | 0.938952 | -0.053135 | 0.0115717 | 4.40E-06 | 62991 | 0.018293254 | 130.3995917 | ukb-b-17406 |

SNP, single nucleotide polymorphism; eaf, effect allele frequency; se, standard error.

**Table S2.** **MR-PRESSO outlier-corrected MR Analysis for vitamin C (ascorbate) and risk of stroke, ischemic stroke**

| Outcomes | Exposure | MR analysis | OR | *p*-value |
| --- | --- | --- | --- | --- |
| Stroke | Vitamin C (ascorbate) | Raw | 0.98 (0.82 - 1.16) | 7.90E-01 |
|  |  | Outlier-corrected | 0.91 (0.82 - 1.02) | 1.50E-01 |
| Ischemic stroke | Vitamin C (ascorbate) | Raw | 0.99 (0.82 - 1.19) | 9.30E-01 |
|  |  | Outlier-corrected | 0.92 (0.81 - 1.04) | 2.30E-01 |

In outlier-corrected MR analysis, we removed rs68344631; OR, odds ratio; MR, Mendelian randomization.

**Table S3. Two-sample Mendelian randomization estimations showing the effects of Vit E (γ-tocopherol) on the risk of stroke and ischemic stroke by removing outlier rs261301**

| Outcomes | Exposure |  | Inverse-variance weighted | |  | Weighted median | |  | MR-Egger | |
| --- | --- | --- | --- | --- | --- | --- | --- | --- | --- | --- |
|  |  |  | OR (95% CI) | *p*-value |  | OR (95% CI) | *p*-value |  | OR (95% CI) | *p*-value |
| Stroke | Vit E (γ-tocopherol) |  | 0.68(0.50-0.93) | 1.53E-02 |  | 0.71(0.50-1.02) | 6.27E-02 |  | 0.82(0.17-3.87) | 0.81 |
| Ischemic | Vit E (γ-tocopherol) |  | 0.66(0.49-0.89) | 6.40E-03 |  | 0.64(0.43-0.95) | 2.63E-02 |  | 0.67(0.15-3.04) | 0.63 |

MR, Mendelian randomization; CI, confidence interval; OR, odds ratio; Vit, vitamin.

**Table S4. IVs trait of dietary antioxidants in PhenoScanner V2：**

| SNP | A1 | A2 | trait | beta | se | *p*-value | sample size |
| --- | --- | --- | --- | --- | --- | --- | --- |
| rs10484100 | A | G | Zinc levels | NA | NA | 3.30E-06 | 2603 |
| rs10484100 | A | G | Blood trace element Zn levels | NA | NA | 3.00E-06 | - |
| rs10931753 | C | G | Zinc levels | NA | NA | 4.94E-06 | 2603 |
| rs10931753 | C | G | Blood trace element Zn levels | NA | NA | 5.00E-06 | - |
| rs11232535 | C | T | Zinc levels | NA | NA | 6.73E-07 | 2603 |
| rs11232535 | C | T | Blood trace element Zn levels | NA | NA | 7.00E-07 | - |
| rs11763353 | A | G | Zinc levels | NA | NA | 6.90E-07 | 2603 |
| rs11763353 | A | G | Blood trace element Zn levels | NA | NA | 7.00E-07 | - |
| rs10484100 | A | G | Zinc levels | NA | NA | 3.30E-06 | 2603 |
| rs10484100 | A | G | Blood trace element Zn levels | NA | NA | 3.00E-06 | - |
| rs10931753 | C | G | Zinc levels | NA | NA | 4.94E-06 | 2603 |
| rs10931753 | C | G | Blood trace element Zn levels | NA | NA | 5.00E-06 | - |
| rs11232535 | C | T | Zinc levels | NA | NA | 6.73E-07 | 2603 |
| rs1532423 | G | A | High light scatter percentage of red cells | 0.02297 | 0.003614 | 2.06E-10 | 173480 |
| rs1532423 | G | A | High light scatter reticulocyte count | 0.02363 | 0.003614 | 6.27E-11 | 173480 |
| rs1532423 | G | A | Immature fraction of reticulocytes | 0.01599 | 0.003579 | 7.84E-06 | 173480 |
| rs1532423 | G | A | Mean corpuscular hemoglobin concentration | 0.01957 | 0.00349 | 2.05E-08 | 173480 |
| rs1532423 | G | A | Reticulocyte count | 0.02292 | 0.00362 | 2.43E-10 | 173480 |
| rs1532423 | G | A | Reticulocyte fraction of red cells | 0.02213 | 0.003618 | 9.53E-10 | 173480 |
| rs1532423 | G | A | Zinc levels | NA | NA | 6.40E-12 | 2603 |
| rs1532423 | G | A | Blood trace element Zn levels | -0.178 | 0.02587 | 6.00E-12 | - |
| rs1532423 | G | A | Reticulocyte fraction of red cells | 0.02213 | 0.003622 | 1.00E-09 | - |
| rs1532423 | G | A | High light scatter percentage of red cells | 0.02297 | 0.003614 | 2.06E-10 | 173480 |
| rs1532423 | G | A | High light scatter reticulocyte count | 0.02363 | 0.003614 | 6.27E-11 | 173480 |
| rs1532423 | G | A | Immature fraction of reticulocytes | 0.01599 | 0.003579 | 7.84E-06 | 173480 |
| rs1532423 | G | A | Mean corpuscular hemoglobin concentration | 0.01957 | 0.00349 | 2.05E-08 | 173480 |
| rs1532423 | G | A | Reticulocyte count | 0.02292 | 0.00362 | 2.43E-10 | 173480 |
| rs1532423 | G | A | Reticulocyte fraction of red cells | 0.02213 | 0.003618 | 9.53E-10 | 173480 |
| rs1532423 | G | A | Zinc levels | NA | NA | 6.40E-12 | 2603 |
| rs1532423 | G | A | Blood trace element Zn levels | -0.178 | 0.02587 | 6.00E-12 | - |
| rs1532423 | G | A | Reticulocyte fraction of red cells | 0.02213 | 0.003622 | 1.00E-09 | - |
| rs2120019 | C | T | Mean corpuscular hemoglobin concentration | 0.0228 | 0.004386 | 2.01E-07 | 173480 |
| rs2120019 | C | T | Mean corpuscular volume | -0.02818 | 0.004473 | 2.99E-10 | 173480 |
| rs2120019 | C | T | White blood cell count | -0.02009 | 0.004541 | 9.71E-06 | 173480 |
| rs2120019 | C | T | Mean corpuscular volume | NA | NA | 2.10E-07 | 71861 |
| rs2120019 | C | T | Zinc levels | NA | NA | 1.55E-18 | 2603 |
| rs2120019 | C | T | Blood trace element Zn levels | 0.287 | 0.03277 | 2.00E-18 | - |
| rs2120019 | C | T | Treatment with amitriptyline | -0.001957 | 0.0004244 | 3.98E-06 | 337159 |
| rs2120019 | C | T | Mean corpuscular hemoglobin concentration | 0.0228 | 0.004386 | 2.01E-07 | 173480 |
| rs2120019 | C | T | Mean corpuscular volume | -0.02818 | 0.004473 | 2.99E-10 | 173480 |
| rs2120019 | C | T | White blood cell count | -0.02009 | 0.004541 | 9.71E-06 | 173480 |
| rs2120019 | C | T | Mean corpuscular volume | NA | NA | 2.10E-07 | 71861 |
| rs2120019 | C | T | Zinc levels | NA | NA | 1.55E-18 | 2603 |
| rs2120019 | C | T | Blood trace element Zn levels | 0.287 | 0.03277 | 2.00E-18 | - |
| rs2120019 | C | T | Treatment with amitriptyline | -0.001957 | 0.0004244 | 3.98E-06 | 337159 |
| rs4333127 | A | G | Zinc levels | NA | NA | 3.00E-06 | 2603 |
| rs4333127 | A | G | Blood trace element Zn levels | NA | NA | 3.00E-06 | - |
| rs7148590 | A | G | Mean corpuscular hemoglobin | 0.06015 | 0.003554 | 2.85E-64 | 173480 |
| rs7148590 | A | G | Mean corpuscular hemoglobin concentration | 0.03474 | 0.003475 | 1.58E-23 | 173480 |
| rs7148590 | A | G | Mean corpuscular volume | 0.04962 | 0.003544 | 1.50E-44 | 173480 |
| rs7148590 | A | G | Mean platelet volume | -0.02042 | 0.003635 | 1.93E-08 | 173480 |
| rs7148590 | A | G | Platelet count | 0.02197 | 0.003662 | 1.96E-09 | 173480 |
| rs7148590 | A | G | Red blood cell count | -0.02654 | 0.003571 | 1.07E-13 | 173480 |
| rs7148590 | A | G | Mean corpuscular hemoglobin concentration | NA | NA | 2.79E-11 | 71861 |
| rs7148590 | A | G | Mean corpuscular volume | NA | NA | 1.29E-08 | 24167 |
| rs7148590 | A | G | Mean corpuscular volume | NA | NA | 6.03E-08 | 71861 |
| rs7148590 | A | G | Zinc levels | NA | NA | 1.37E-07 | 2603 |
| rs7148590 | A | G | Blood trace element Zn levels | NA | NA | 1.00E-07 | - |
| rs7148590 | A | G | Comparative height size at age 10 | -0.01011 | 0.001661 | 1.15E-09 | 332021 |
| rs7148590 | A | G | Hand grip strength left | -0.00878 | 0.001755 | 5.63E-07 | 335821 |
| rs7148590 | A | G | Hand grip strength right | -0.008792 | 0.001758 | 5.70E-07 | 335842 |
| rs7148590 | A | G | Height | -0.01448 | 0.001729 | 5.54E-17 | 336474 |
| rs7148590 | A | G | Sitting height | -0.01233 | 0.001881 | 5.58E-11 | 336172 |
| rs7148590 | A | G | Trunk fat-free mass | -0.008148 | 0.001533 | 1.07E-07 | 331030 |
| rs7148590 | A | G | Trunk predicted mass | -0.007975 | 0.001528 | 1.80E-07 | 330995 |
| rs7148590 | A | G | Whole body fat-free mass | -0.007217 | 0.001539 | 2.75E-06 | 331291 |
| rs7148590 | A | G | Whole body water mass | -0.006958 | 0.001541 | 6.35E-06 | 331315 |
| rs7148590 | A | G | Mean corpuscular hemoglobin | 0.06015 | 0.003554 | 2.85E-64 | 173480 |
| rs7148590 | A | G | Mean corpuscular hemoglobin concentration | 0.03474 | 0.003475 | 1.58E-23 | 173480 |
| rs7148590 | A | G | Mean corpuscular volume | 0.04962 | 0.003544 | 1.50E-44 | 173480 |
| rs7148590 | A | G | Mean platelet volume | -0.02042 | 0.003635 | 1.93E-08 | 173480 |
| rs7148590 | A | G | Platelet count | 0.02197 | 0.003662 | 1.96E-09 | 173480 |
| rs7148590 | A | G | Red blood cell count | -0.02654 | 0.003571 | 1.07E-13 | 173480 |
| rs7148590 | A | G | Mean corpuscular hemoglobin concentration | NA | NA | 2.79E-11 | 71861 |
| rs7148590 | A | G | Mean corpuscular volume | NA | NA | 1.29E-08 | 24167 |
| rs7148590 | A | G | Mean corpuscular volume | NA | NA | 6.03E-08 | 71861 |
| rs7148590 | A | G | Zinc levels | NA | NA | 1.37E-07 | 2603 |
| rs7148590 | A | G | Blood trace element Zn levels | NA | NA | 1.00E-07 | - |
| rs7148590 | A | G | Comparative height size at age 10 | -0.01011 | 0.001661 | 1.15E-09 | 332021 |
| rs7148590 | A | G | Hand grip strength left | -0.00878 | 0.001755 | 5.63E-07 | 335821 |
| rs7148590 | A | G | Hand grip strength right | -0.008792 | 0.001758 | 5.70E-07 | 335842 |
| rs7148590 | A | G | Height | -0.01448 | 0.001729 | 5.54E-17 | 336474 |
| rs7148590 | A | G | Sitting height | -0.01233 | 0.001881 | 5.58E-11 | 336172 |
| rs7148590 | A | G | Trunk fat-free mass | -0.008148 | 0.001533 | 1.07E-07 | 331030 |
| rs7148590 | A | G | Trunk predicted mass | -0.007975 | 0.001528 | 1.80E-07 | 330995 |
| rs7148590 | A | G | Whole body fat-free mass | -0.007217 | 0.001539 | 2.75E-06 | 331291 |
| rs7148590 | A | G | Whole body water mass | -0.006958 | 0.001541 | 6.35E-06 | 331315 |
| rs7148590 | A | G | Mean corpuscular hemoglobin | 0.06015 | 0.003554 | 2.85E-64 | 173480 |
| rs7148590 | A | G | Mean corpuscular hemoglobin concentration | 0.03474 | 0.003475 | 1.58E-23 | 173480 |
| rs7148590 | A | G | Mean corpuscular volume | 0.04962 | 0.003544 | 1.50E-44 | 173480 |
| rs7148590 | A | G | Mean platelet volume | -0.02042 | 0.003635 | 1.93E-08 | 173480 |
| rs7148590 | A | G | Platelet count | 0.02197 | 0.003662 | 1.96E-09 | 173480 |
| rs7148590 | A | G | Red blood cell count | -0.02654 | 0.003571 | 1.07E-13 | 173480 |
| rs7148590 | A | G | Mean corpuscular hemoglobin concentration | NA | NA | 2.79E-11 | 71861 |
| rs7148590 | A | G | Mean corpuscular volume | NA | NA | 1.29E-08 | 24167 |
| rs7148590 | A | G | Mean corpuscular volume | NA | NA | 6.03E-08 | 71861 |
| rs7148590 | A | G | Zinc levels | NA | NA | 1.37E-07 | 2603 |
| rs7148590 | A | G | Blood trace element Zn levels | NA | NA | 1.00E-07 | - |
| rs7148590 | A | G | Comparative height size at age 10 | -0.01011 | 0.001661 | 1.15E-09 | 332021 |
| rs7148590 | A | G | Hand grip strength left | -0.00878 | 0.001755 | 5.63E-07 | 335821 |
| rs7148590 | A | G | Hand grip strength right | -0.008792 | 0.001758 | 5.70E-07 | 335842 |
| rs7148590 | A | G | Height | -0.01448 | 0.001729 | 5.54E-17 | 336474 |
| rs7148590 | A | G | Sitting height | -0.01233 | 0.001881 | 5.58E-11 | 336172 |
| rs7148590 | A | G | Trunk fat-free mass | -0.008148 | 0.001533 | 1.07E-07 | 331030 |
| rs7148590 | A | G | Trunk predicted mass | -0.007975 | 0.001528 | 1.80E-07 | 330995 |
| rs7148590 | A | G | Whole body fat-free mass | -0.007217 | 0.001539 | 2.75E-06 | 331291 |
| rs7148590 | A | G | Whole body water mass | -0.006958 | 0.001541 | 6.35E-06 | 331315 |
| rs3785832 | T | C | Height | -0.008531 | 0.001763 | 1.32E-06 | 336474 |
| rs7163368 | C | T | Reticulocyte count | 0.02002 | 0.004378 | 4.80E-06 | 173480 |
| rs7163368 | C | T | Cause of death: heart failure, unspecified | 0.002952 | 0.0006414 | 4.23E-06 | 7637 |
| rs7700970 | C | T | Selenium levels | NA | NA | 1.72E-18 | 2603 |
| rs7700970 | C | T | Toenail selenium levels | -0.03 | 0.004474 | 2.00E-11 | - |
| rs7700970 | C | T | Selenium levels | NA | NA | 1.72E-18 | 2603 |
| rs7700970 | C | T | Toenail selenium levels | -0.03 | 0.004474 | 2.00E-11 | - |
| rs10245705 | C | T | Cause of death: calculus of gallbladder without cholecystitis | -0.01037 | 0.00213 | 1.14E-06 | 7637 |
| rs2070006 | T | C | Gamma fibrinogen levels | NA | NA | 4.60E-30 | 3042 |
| rs2070006 | T | C | Venous thrombosis | NA | NA | 6.05E-06 | 2652 |
| rs2070006 | T | C | Blood clot in the leg | 0.001736 | 0.0003563 | 1.09E-06 | 336782 |
| rs2070006 | T | C | Blood clot in the lung | 0.001315 | 0.0002278 | 7.90E-09 | 336782 |
| rs2070006 | T | C | Phlebitis and thrombophlebitis | 0.0008405 | 0.0001734 | 1.24E-06 | 337199 |
| rs2070006 | T | C | Pulmonary embolism | 0.0008068 | 0.0001507 | 8.68E-08 | 337199 |
| rs2070006 | T | C | Self-reported deep venous thrombosis | 0.001702 | 0.0003524 | 1.37E-06 | 337159 |
| rs2070006 | T | C | Self-reported pulmonary embolism + or - dvt | 0.001284 | 0.0002281 | 1.83E-08 | 337159 |
| rs6826474 | C | T | Cause of death: congestive heart failure | -0.008902 | 0.001831 | 1.18E-06 | 7637 |
| rs7112460 | C | T | Mean platelet volume | 0.0334 | 0.007412 | 6.59E-06 | 173480 |
| rs7112460 | C | T | CD8mem:%pre-Th17 (1); CD8 pre Th17 (CD161+PD1-) | 0.1958 | 0.04401 | 8.61E-06 | 427 |
| rs7112460 | C | T | CD8mem:%pre-Th17 (2); CD8 preTh17 (CD161+) | 0.196 | 0.04412 | 8.86E-06 | 428 |
| rs1060467 | G | A | Circulating a tocopherol concentrations following 3 y of controlled vitamin E supplementation in men | NA | NA | 1.60E-06 | 2112 |
| **rs261301*** | T | C | High density lipoprotein | 0.0808 | 0.0072 | 4.39E-24 | 92803 |
| **rs261301*** | T | C | High density lipoprotein | 0.0772 | 0.0073 | 6.14E-25 | 99900 |
| **rs261301*** | T | C | HDL cholesterol | NA | NA | 6.14E-25 | 100184 |
| **rs261301*** | T | C | High density lipoprotein | 0.094 | 0.0086 | 4.62E-28 | 34370 |
| **rs261301*** | T | C | Total cholesterol | 0.0436 | 0.0091 | 1.79E-06 | 32014 |
| rs17800766 | C | T | Treatment with nitrofurantoin | 0.001206 | 0.0002671 | 6.36E-06 | 337159 |
| rs4771831 | A | G | Height | NA | NA | 9.69E-07 | 129476 |
| rs4771831 | A | G | Height | -0.02528 | 0.004865 | 2.03E-07 | 129315 |
| rs4771831 | A | G | Height | -0.017 | 0.0033 | 2.00E-07 | 247997 |
| rs4771831 | A | G | Height | NA | NA | 9.69E-07 | 133653 |
| rs4771831 | A | G | Comparative height size at age 10 | -0.008927 | 0.001744 | 3.09E-07 | 332021 |
| rs4771831 | A | G | Height | -0.01002 | 0.001815 | 3.37E-08 | 336474 |
| rs6596473 | C | G | Height | 0.009607 | 0.001895 | 3.97E-07 | 336474 |
| rs6596473 | C | G | Overall health rating | -0.01014 | 0.001936 | 1.63E-07 | 336020 |
| rs6596473 | C | G | Pulse rate | -0.01221 | 0.002746 | 8.78E-06 | 317756 |
| rs6596473 | C | G | Sitting height | 0.009305 | 0.002061 | 6.35E-06 | 336172 |
| rs6596473 | C | G | Time spent watching television | -0.01002 | 0.002052 | 1.04E-06 | 319740 |
| rs6596473 | C | G | Trunk fat-free mass | 0.007806 | 0.00168 | 3.37E-06 | 331030 |
| rs6596473 | C | G | Trunk predicted mass | 0.007775 | 0.001674 | 3.43E-06 | 330995 |
| rs6596473 | C | G | Height | 0.009607 | 0.001895 | 3.97E-07 | 336474 |
| rs6596473 | C | G | Overall health rating | -0.01014 | 0.001936 | 1.63E-07 | 336020 |
| rs6596473 | C | G | Pulse rate | -0.01221 | 0.002746 | 8.78E-06 | 317756 |
| rs6596473 | C | G | Sitting height | 0.009305 | 0.002061 | 6.35E-06 | 336172 |
| rs6596473 | C | G | Time spent watching television | -0.01002 | 0.002052 | 1.04E-06 | 319740 |
| rs6596473 | C | G | Trunk fat-free mass | 0.007806 | 0.00168 | 3.37E-06 | 331030 |
| rs6596473 | C | G | Trunk predicted mass | 0.007775 | 0.001674 | 3.43E-06 | 330995 |
| rs117669768 | A | G | Unspecified nephritic syndrome | 0.0004167 | 9.42E-05 | 9.68E-06 | 337199 |
| rs692790 | C | T | Immature fraction of reticulocytes | 0.03421 | 0.005299 | 1.08E-10 | 173480 |

* rs261301 (an IV of γ-tocopherol) was associated with high-density lipoprotein and cholesterol. NA, Not Applicable; HDL, High Density Lipoprotein; A1, effect allele; A2, other allele; se, standard error
